# Comparison of medetomidine, thiopental and ketamine/midazolam anesthesia in chick embryos for *in ovo* Magnetic Resonance Imaging free of motion artifacts

**DOI:** 10.1038/srep15536

**Published:** 2015-10-23

**Authors:** Conny Waschkies, Flora Nicholls, Johanna Buschmann

**Affiliations:** 1 Institute for Biomedical Engineering, ETH and University of Zurich, HIT E22.3 Wolfgang-Pauli-Str. 27, 8093 Zurich, Switzerland; 2 Visceral and Transplant Surgery, University Hospital Zurich, Sternwartstr. 14, 8091 Zurich, Switzerland; 3 BZL, Biological Central Laboratory, University Hospital Zurich, Sternwartstrasse 6, 8091 Zurich, Switzerland; 4 Plastic Surgery and Hand Surgery, University Hospital Zurich, Sternwartstr. 14, 8091 Zurich, Switzerland

## Abstract

Non-invasive assessment of the perfusion capacity of tissue engineered constructs grown on the chorioallantoic membrane by MRI is often hampered by motion artifacts. Therefore, we examined the suitability of three anesthetic regimes for sufficient sedation of the chick embryo. Medetomidine at a dosage of 0.3 mg/kg, was compared to thiopental at 100 mg/kg and ketamine/midazolam at 50 mg/kg and 1 mg/kg, respectively. These soluble anesthetics were applied by dropping a total volume of 0.3 mL onto the surface of the CAM. Motion was videotaped through the window of the eggshell and scored semi-quantitatively. Medetomidine performed best in terms of reduced motion; onset of anesthesia occurred within 10 minutes and for the following 30 minutes, allowing proper *in vivo* MRI measurements. The other regimen were not sedating deep enough (ketamine/midazolam) and not long enough (thiopental). In sum, medetomidine allows proper sedation for MRI assessment of the perfusion capacity in a tissue engineered construct placed on the CAM.

The chorioallantoic membrane (CAM) of the chick embryo is a well-established model for the study of vascularization *in vivo* and is typically referred to as CAM assay^1^,^2^. It is a very useful model because of the easy accessibility of the vascular network. Moreover, its lack of immune competence enables studies dealing with tumor growth, wound healing, angiogenesis, anti-angiogenesis or biocompatibility of xenografts^3^,^4^,^5^. In addition, the CAM provides an excellent environment where the cells can attach and proliferate in 3D scaffolds and biomaterials. We recently developed a new method to non-invasively monitor the vascularization of implanted biomaterial scaffolds on the CAM assay in order to assess the perfusion capacities of such implants and hence their success of tissue regeneration *in vivo*^6^. This method was based on *in vivo* Magnetic Resonance Imaging (MRI).

In order to obtain reliable, high-quality MR images and to produce quantitative maps of perfusion capacity from biomaterial scaffolds grown on the CAM of the chick embryo, motion of the chick embryo needs to be minimal. For this purpose we applied in our previous study 5 drops of 1:100 M ketamine (25 mg/kg) onto the CAM in order to sedate the chick embryo^6^. However, motion of the chick embryo, and hence the scaffold planted on its CAM, was still observed in about 20% of our samples, which consequently, had to be excluded from analysis due to motion artifacts (Fig. 1a,b). Obviously, a more effective anesthesia/sedation protocol that provides stable sedation of the chick embryo for a total of about 1h would be highly appreciated. Pre- and post-contrast MR measurements last about 30 min each, and a second dosage may be given between these two imaging blocks. As access to physiological parameters such as cardiac frequency and blood pressure is limited in the chicken embryo, depths of anesthesia can neither be precisely monitored nor modulated, calling for a robust, yet simple-to-apply-and-maintain anesthesia protocol.

Only few studies so far have investigated sedation of chick embryos *in ovo*, mostly in the context of *in vivo* imaging. For example, chick embryos were studied *in ovo* using MRI during development^7^,^8^ using mild cooling conditions for sedation. Other studies applied ketamine anesthesia during MRI for characterization of embryonic tissue development^9^ or in cell tracking studies in the living chick embryo^10^ Also inhalant anesthetics were employed such as halothane and isoflurane delivered through the egg shell^11^,^12^,^13^. These however required more handling and specific equipment, such as sealable plastic bags to maintain the egg under anesthesia atmosphere, as well as dedicated vaporizers. Therefore, we explored the soluble anesthesia classes of narcotics for better suited, effective and easy-to-use anesthesia protocols for our specific purpose.

We examined three anesthetic regimen from different substance classes: i.e. medetomidine as an alpha-2-adrenoreceptor agonist; thiopental, a barbiturate; and the combination anesthesia ketamine/midazolam with ketamine acting primarily as a NMDA receptor antagonist and midazolam belonging to the benzodiazepine class of sedatives. In sum, the objective of the present study was to compare three anesthetic regimen in the chick embryo with the purpose to provide easy sedation that enables the non-invasive assessment of perfusion capacity in biomaterials grown on the CAM by contrast-enhanced MRI^6^.

## Materials and Methods

### CAM assay

Fertilized Lowman white LSL chick eggs (Animalco AG Geflügelzucht, Staufen, Switzerland) were incubated under sterile conditions at 37 °C and 65% relative humidity. On incubation day (ID) 3.5 a circular window with a diameter of 40–45 mm was drilled into the eggshell after removing 2 mL albumen so that the developing chorioallantoic membrane detached from the eggshell. The window in the eggshell was closed with a sterile Petri dish of 50 mm to prevent dehydration. Afterwards the eggs were incubated until ID 14. According to Swiss animal care guidelines, no IACUC approval was necessary to perform the experiments in chick embryo experiments. According to the local guidelines, only experiments with chick embryos older than embryonic day 15 need IACUC approval. However, the embryos used in this study were all in earlier stages of embryonic development.

### Anesthesia

Three different anesthesia regimen were tested in our study, with n = 7 for each regimen. The dosage was established based on a literature search^14^,^15^,^16^ and pilot experiments were performed where the highest below lethal dosage was assessed. Anesthesia solutions were dropped directly onto the CAM at an effective application volume of 0.3 mL, resulting in good coating of and uptake through the CAM.

#### Medetomidine anesthesia

Medetomidine was purchased from Dr. E. Graeub AG, Bern, Switzerland (Dorbene® *ad us. vet*., Injektionslösung). The stock solution (1 mg/mL) was diluted to 1:100 in saline (0.9% NaCl, Braun B. Medical AG, Switzerland) and applied to the CAM of the chick embryo at a dosage of 0.3 mg/kg in 0.3 ml application volume. As an antagonist atipamezol (Alzane®, *ad us. vet*., Dr. E. Graeub AG, Bern, Switzerland) was given at a dosage of 0.3 mg/kg after one hour of anesthesia. For that purpose, the stock solution (5 mg/mL) was diluted to 1:500 and 0.3 mL dropped on the CAM.

#### Thiopental anesthesia

Thiopental was bought in 1 g vials from Ospedalia AG, Switzerland. 1.0 g of the powder was dissolved in 10 mL saline, then further diluted down to 1:30 and 0.3 mL solution dropped on the CAM to provide a dosage of 100 mg/kg.

#### Ketamine/Midazolam anesthesia

Ketasol-100 was purchased from Graeub, Switzerland. For a dosage of 50 mg/kg a 1:30 dilution was prepared from stock solution and 0.15 mL ketamine were mixed with 0.15 mL midazolam (Dormicum®, Graeub, Switzerland), which was diluted to 1:75 in saline from stock solution of 5 mg/mL, resulting in a dosage of 1 mg/kg. After one hour midazolam was antagonized with flumazenil (Anexate® Alloga AG, Roche Pharma), at 0.1 mg/kg dosage, diluted 1:30 from stock solution of 0.1ml/ml resulting in an application volume of 0.3 ml.

### Motion analysis

All anesthesia experiments were performed on ID 14, the day the vasculature developed is highest before IACUC approval is needed, and hence a typical time point used in experiments with CAM assays. Motion of the chick embryos was videotaped through the window in the eggshell repetitively for periods of 4 min prior anesthesia, and in 10 min intervals for one hour after anesthesia, and immediately after antagonization. In order to study the sedative effect of the different anesthesia regimen under conditions similar to that of the MRI machine, loud sound recorded from a MRI measurement was replayed during the videotaping periods. Throughout the experiments, eggs were maintained on a warming plate and were covered by a dark box when not videotaped. Severity of motion was scored qualitatively with **0** no detectable movement, **1** slight movement that might still be acceptable for MRI of biomaterial samples placed on the CAM, such as smooth small movement of the chick embryo beneath the CAM surface that does not disturb the scaffold on the CAM, **2** gross gliding motion or “kicking” that is judged to prevent artifact-free MRI of samples placed on the CAM and **3** gross gliding motion and “kicking” of the chick embryo (Supporting Information videos of each score).

### Statistical analysis

The data were analyzed with StatView 5.0.1 software. One-way statistical analysis of variance (ANOVA) was conducted to test the significance of differences between either the time points of the same anesthetic or between the three anesthetic regimen at one time point. Pairwise comparison probabilities (*p*) were calculated using the Fisher’s PLSD post hoc test to evaluate differences between the groups. *P* values < 0.05 were considered significant. Values are expressed as means ± standard errors.

## Results

### Comparison of three anesthesia regimen

**Figure 2:**
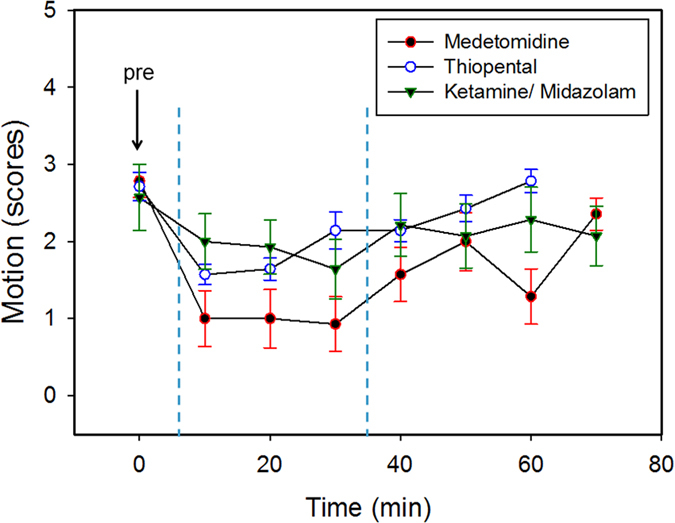
Motion scores of chick embryo movement as a function of time for three anesthesia regimen, medetomidine at 0.3 mg/kg, ketamine/midazolam at 50 mg/kg and 1 mg/kg, and thiopental at 100 mg/kg, respectively (n = 7). Scores were assigned during each 4 min of videotaping, immediately prior anesthesia (0 min), and every 10 min during anesthesia for one hour and immediately after antagonization where applicable (70 min). Medetomidine is considered the most suitable of the different anesthesia regimen studied for the purpose to assess perfusion capacity of biomaterials grown on the CAM non-destructively *in ovo* using contrast-enhanced MRI and during the required time frame of 30 minutes (dashed blue lines). Error bars denote standard errors.

When medetomidine, thiopental and ketamine/midazolam were compared with respect to the onset of anesthesia, all three regimen were effective within the first ten minutes after application as observed in reduced motion of the chick embryos and illustrated in lower motion scores (Fig. 2). Obviously, medetomidine generated the deepest sedation of the three regimen with the lowest motion scores, which were maintained for around 30 min. In detail, motion scores under medetomidine anesthesia were significantly lower than the motion scores found for ketamine/midazolam at 10 min (*p* = 0.0324), 20 min (*p* = 0.0480), 30 min (*p* = 0.0188) and 60 min (*p* = 0.0049). Importantly, medetomidine anesthesia reduced motion of the chick embryos to levels considered suitable for MRI. In contrast, thiopental seems quite short-acting for only about 20 min (being significantly lower at time points 10 and 20 min when compared to later time points of thiopental sedation), while ketamine/midazolam anesthesia follows a time course similar to medetomidine, with a slower onset (being not significantly different at any time points measured for ketamine/midazolam). Therefore, both regimen, ketamine/midazolam and thiopental, were only partially effective to reduce motion, with the chick embryos not sedated enough and not long enough to allow for successful MRI. Finally, all chick embryos survived the selected anesthesia regimen for a follow up period of about 20 h.

## Discussion

In the current study, we present a comparison of three anesthesia regimen for sedation of the chick embryo in the windowed egg. The specific goal was to use the CAM assay to assess perfusion capacity in biomaterial scaffolds *in ovo* in the living chick embryo using non-destructive contrast-enhanced MRI devoid of motion artifacts. Of the three anesthesia regimen studied, i.e. medetomidine, thiopental and ketamine/midazolam dropped directly onto the chorioallantoic membrane, medetomidine anesthesia provided the deepest sedation of the chick embryo allowing for MRI undisturbed by motion-artifacts and was effective over a period of 30 min, which is required to complete one MRI session. Hence, medetomidine provided the most adequate anesthesia for our specific purpose.

Vascularization in three-dimensional biomaterials is a limiting factor for the successful implantation and functionalization of tissue engineered constructs. The highly vascularized CAM of the chick embryo is a very suitable model to evaluate the perfusion capacity of scaffolds placed on top of the CAM^2^. It also allows to work with human tumor cells in order to study tumor-induced angiogenesis^17^ or chemoprevention^18^. Importantly, the CAM assay is well established^19^, low priced and predominantly used to study angiogenesis and anti-angiogenesis in response to different drugs, growth factors and cell types in 3D scaffolds placed on the chorioallantoic membrane^3^,^6^,^20^. Contrast-enhanced MRI offers a non-destructive, *in situ*, quantitative tool to monitor the perfusion capacity in biomaterials placed on the CAM. An essential prerequisite, however, is that the chick embryo does not move during the MRI measurement to produce quantitative MRI perfusion maps. Anesthesia/sedation of chick embryos has received some attention over the last years, in particular as non-invasive *in vivo* imaging techniques become more and more available, triggering interest in suitable anesthesia protocols for commensurate applications. In the few studies conducted so far, mild cooling^7^,^8^, ketamine anesthesia^9^,^10^,^21^ and isoflurane^11^,^12^ received most attention. As eggs need to be windowed for our studies for planting tissue engineered constructs onto the CAM, the CAM is easily accessible for soluble anesthetics and hence medetomidine and thiopental were included into our investigations as members of different substance classes not studied so far.

Medetomidine is an alpha-2 adrenoceptor agonist and is used in veterinary medicine as a sedative with muscle relaxant properties^22^. Its effects can be reversed almost immediately by alpha-2 antagonists, and it is not irritating when administered *i.m*. or *i.p*., presumably also when applied directly onto the CAM in our experiments. Medetomidine anesthesia in our study resulted in only slight and slow, gliding movements of the chick embryo, in contrast to the gross and kicking motions observed in the same embryos prior anesthesia. Furthermore, the pharmacokinetics of medetomidine was favorable, providing sedation of the embryo for about 30 min, well suited to the time window used in our MRI investigations. Medetomidine was also used successfully in previous studies, though some behavioral teratogenic effects in chicks following *in ovo* injection^23^ have been described in this particular case, which however, were studied only when applied early during development (ID 4). Taken together, of the anesthesia regimen investigated, medetomidine served as the best-suited anesthesia protocol for the specific purpose of monitoring the perfusion capacity in 3D scaffolds grown on the CAM *in vivo* by MRI.

Barbiturates such as thiopental act as GABA_A_ agonists and are generally considered as good, fast-acting anesthetics; however sedation may not be reliable at the lower dosage range, consistent with our observations. As no obvious advantage over the other substance classes emerged in our study, we consider thiopental as unsuitable for use in the avian embryo; the more so as no effective antagonist is available.

In the third anesthesia protocol, we combined ketamine with midazolame in order to suppress the residual movements that were observed under ketamine-only conditions used in our previous study^6^. Ketamine has been widely applied as sedation narcotic, not only in cockerels with different premedications such as xylazine, diazepam or midazolam^24^, but also in chick embryos for MRI measurements *in ovo*^9^,^10^,^21^. Its wide safety margin is clearly advantageous in particular for less well-studied species and its poor muscle relaxation can be mitigated by combination anesthesia, in our study midazolam. Midazolam is a benzodiazepine often used as an anticonvulsant and sedative in combination anesthesia. It is water soluble and can be antagonized. While a sedative effect was clearly observed in our study, residual motion of the chick embryo was not judged sufficient for MRI, as gross motion and strong kicks were still persistent throughout the experiment.

Finally, it should be noted that all sedatives/anesthetics, also hypothermia as applied in previous studies, inadvertently have systemic effects on the cardiovascular system, such as on heart rate, blood pressure and vascular resistance^25^ which affect any vascular readout. We presume that the three anesthetics used may differ in their impact on perfusion capacity, as assessed with contrast-enhanced MRI. Nevertheless, when the same anesthetic is used at the same dosage for all chick embryos, valid comparisons of perfusion capacity can be made.

In sum, we report the comparison of three anesthetic regimen, medetomidine, thiopental and ketamine/midazolam, all applied directly onto the CAM, in order to sedate the chick embryo for MRI devoid of motion artifacts. Clearly, medetomidine was found suited best for this purpose.

## Additional Information

**How to cite this article**: Waschkies, C. *et al.* Comparison of medetomidine, thiopental and ketamine/midazolam anesthesia in chick embryos for *in ovo* Magnetic Resonance Imaging free of motion artifacts. *Sci. Rep.* **5**, 15536; doi: 10.1038/srep15536 (2015).

## Supplementary Material

Supplementary Information video legends

Supplementary Information score 0

Supplementary Information score 1

Supplementary Information score 2

Supplementary Information score 3

## Figures and Tables

**Figure 1:**
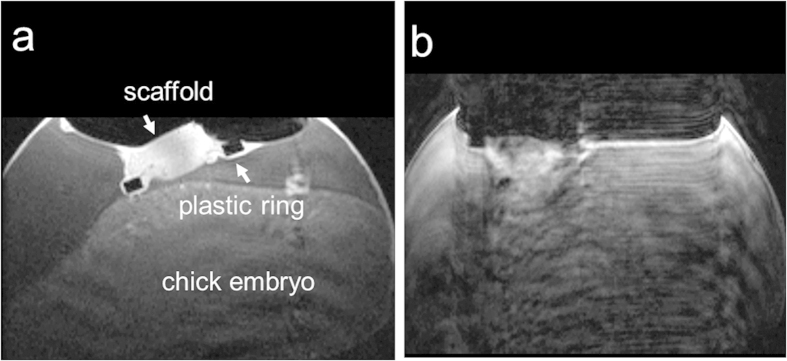
Two *in ovo* T1-weighted MR images of the chick embryo on ID 14, seven days post-implantation on the CAM acquired in an axial slice through the biomaterial scaffold; (a) image without motion artifact and (b) image with motion artifacts that was excluded from further analysis.
