# Supplementary material for: Comparison of medetomidine, thiopental and ketamine/midazolam anesthesia in chick embryos for in ovo Magnetic Resonance Imaging free of motion artifacts
Source: Sci Rep. 2015 Oct 23;5:15536. doi: 10.1038/srep15536 (PMC4616043; doi:10.1038/srep15536)
Supplement: Supplementary Information video legends [file srep15536-s1.pdf]

Supplementary Information: Legends to the videos

Comparison of medetomidine, thiopental and ketamine/midazolam anesthesia in chick embryos for *in ovo* Magnetic Resonance Imaging free of motion artifacts

Conny Waschkies, PhD[1,2], Flora Nicholls[3] and Johanna Buschmann, PhD[4*]

[1]Institute for Biomedical Engineering, ETH and University of Zurich, HIT E22.3 Wolfgang-Pauli-Str. 27, 8093 Zurich, Switzerland

[2]Visceral and Transplant Surgery, University Hospital Zurich, Sternwartstr. 14, 8091 Zurich, Switzerland

[3]BZL, Biological Central Laboratory, University Hospital Zurich, Sternwartstrasse 6, 8091 Zurich, Switzerland

[4]Plastic Surgery and Hand Surgery, University Hospital Zurich, Sternwartstr. 14, 8091 Zurich, Switzerland

*corresponding author: johanna.buschmann@usz.ch

Dr. Johanna Buschmann

University Hospital Zurich

Plastic Surgery and Hand Surgery

Sternwartstrasse 14

8091 Zürich

Legend to the video "score0. mov"

In this video the motion of the chick embryo was assessed when the movements were not detectable (score = 0).

Legend to the video "score1. mov"

In this video the motion of the chick embryo was assessed when there was a slight movement of the embryo that might still be acceptable for MRI of biomaterial samples placed on the CAM, such as smooth small movement of the chick embryo beneath the CAM surface that does not disturb the scaffold on the CAM (score = 1).

Legend to the video "score2. mov"

In this video the motion of the chick embryo was assessed when there was a gross gliding motion or "kicking" that was judged to prevent artifact-free MRI of samples placed on the CAM (score = 2).

Legend to the video "score3. mov"

In this video the motion of the chick embryo was assessed during gross gliding motion and "kicking" of the chick embryo (score = 3).
